# Research priorities in psychiatric genetic counselling: how to talk to children and adolescents about genetics and psychiatric disorders

**DOI:** 10.1038/s41431-022-01253-0

**Published:** 2022-12-21

**Authors:** Jessica Mundy, Helena L. Davies, Mădălina Radu, Jehannine Austin, Evangelos Vassos, Thalia C. Eley, Gerome Breen, Ramona Moldovan

**Affiliations:** 1 Social, Genetic and Developmental Psychiatry Centre, Institute of Psychiatry, Psychology and Neuroscience, King’s College London, London, UK; 2 Department of Psychology, Babeş-Bolyai University, Cluj-Napoca, Romania; 3 Departments of Psychiatry and Medical Genetics, The University of British Columbia, Vancouver, BC Canada; 4 BC Mental Health and Substance Use Services Research Institute, Vancouver, BC Canada; 5 UK National Institute for Health Research (NIHR) Biomedical Research Centre for Mental Health, South London and Maudsley Hospital, London, UK; 6 Division of Evolution and Genomic Sciences, School of Biological Science, University of Manchester, Manchester, UK; 7 Manchester Centre for Genomic Medicine, St Mary’s Hospital, Manchester University Hospitals NHS Foundation Trust, Manchester Academic Health Science Centre, Manchester, UK

**Keywords:** Social sciences, Patient education, Public health, Health policy

## Background

It is now well established that mental health disorders are heritable [1]. Genetic counselling is a process through which a trained professional helps an individual to better understand and adapt to the medical, psychological, and familial implications of genetic contributions to disease [2]. As of yet, no genetic tests to confirm a psychiatric diagnosis exist and alone they are unlikely to be sufficient for diagnosis. However, a formal genetic test is not required for the delivery of genetic counselling and for the benefits to be realised [3]. Psychiatric genetic counselling is conceptually identical to genetic counselling for other types of disorders and its efficacy for adults diagnosed with psychiatric disorders and their family members has been documented [4]. We are now entering an era in which genetic information is likely to become far more available as it is gradually integrated into healthcare. Thus, how to effectively communicate complex genetic risk information is of high research priority.

Communicating genetic risk for psychiatric disorders comes with many challenges due to their complex and multifactorial nature. Nonetheless, psychiatric genetic counselling is associated with a number of positive immediate and long-term outcomes [4]. For instance, psychiatric genetic counselling can tackle misconceptions about causes of illness, address genetic and/or environmental determinism, empower and reduce shame and/or guilt, change one’s approach to treatment, and enable more informed decision-making regarding major life decisions, such as having children [4]. Such established benefits suggest that psychiatric genetic counselling will become an important part of clinical care for psychiatric patients in the future.

Half of mental health disorders start before the age of 14 [5], with 1 in 7 young people between 10 to 19 years old experiencing mental ill health [6]. Thus, childhood or adolescence could be a particularly suitable window within which to receive psychiatric genetic counselling. This may prevent misconceptions about the causes of one’s mental illness, manage stigmatising beliefs related to personal or family history of mental health problems [7], and encourage risk-reducing behaviours [8, 9]. Psychiatric genetic counselling could also have a positive impact on parents and caregivers, who often feel responsible for their child’s mental health and may experience feelings of guilt, shame, or a heavy burden of responsibility [10]. Such feelings may be partially rooted in a limited understanding of the contributions of genetic and environmental factors to mental disorders [11]. This can have a variety of negative behavioural consequences such as not seeking out suitable support for their child or potentially limiting the number of their future children [12, 13].

## Lack of research into the communication of genetic risk information to children and adolescents

Research exploring physical health conditions in young people highlights a strong desire to better understand the cause/s of their diagnosed disorder [14, 15]. Given the complex nature of psychiatric disorders, genetic counsellors recognise that children and adolescents require specific tools and techniques to adequately comprehend information about the aetiology and management of their condition. For example, adapting the wording of medical terminology or using metaphors and visual aids may prove beneficial [16]. A mixed-method systematic review analysing the literature on genetic counselling for children and adolescents identified a dearth of publications on how best to communicate this type of information to children and adolescents (Radu M, Moldovan R. Genetic counseling for children and adolescents: A mixed-method systematic review. 2022. Manuscript in preparation.). The authors noted the stark contrast between the large number of guidelines and recommendations available and the relatively scarce number of empirical research studies. The picture is even less clear for psychiatric disorders. Furthermore, to this day, studies investigating the efficacy of psychiatric genetic counselling have focussed on adults only [17].

## Research directions and practical recommendations

We propose the following research priorities:

1. What is the most effective way to communicate personal genetic risk to children and adolescents?
  Young people form a distinct patient group and may need specific strategies or tools to explain the concept of inherited genetic risk for psychiatric disorders. For instance, the ‘mental health jar’ has proved to be extremely successful within adults with psychiatric disorders [8]. However, traditional communication styles used with adults may not be directly translatable to young people. Research now needs to investigate whether this tool (and others) are similarly successful in young populations. Furthermore, given that genetic information may have implications for other family members, genetic counsellors can support individuals when they are adapting to the medical, psychological, and familial implications of their condition [17].
2. When is the most appropriate time to discuss risk for psychiatric disorders with young people?
  Young people want to learn about their physical disorders at a much younger age than when genetic counselling is routinely offered [14, 15]. As most research has focused on physical health conditions or on adults with psychiatric conditions, the effectiveness of psychiatric genetic counselling in younger age groups is still unclear. Further, *c*ommunication requirements may differ depending on the specific developmental stage within childhood and adolescence, or indeed the psychiatric problems experienced. A recent qualitative study explored the opinions of ten parents of children with the genetic condition 22q11.2 deletion syndrome and highlighted that the child’s developmental age and thus ability to comprehend complex information influenced when they decided to initiate discussions about the cause of the disorder [18]. Similar types of research are required to establish the optimal age period within childhood and/or adolescence during which psychiatric genetic counselling is most effective for a range of disorders.
3. Who is best placed to offer this service?
  Given the prevalence of mental illnesses and the relative dearth of genetic counsellors (which falls far below the recommendations by the WHO), a focused effort to train other healthcare professionals may reduce reliance on genetic counsellors. A meta-analysis found that the type of professional, i.e., genetic counsellors versus other specialists, had no significant impact on the association between psychiatric genetic counselling and positive outcomes in adults, such as reductions in anxiety or guilt and increase in empowerment or self-efficacy [4]. However, more research is needed to understand the ability of other specialists to fulfil this role effectively within child and adolescent services after adequate training. Some argue that all medical professionals who interact with psychiatric patients may be required to initiate discussions about genetic and environmental risk. As such, there is an argument for mainstreaming psychiatric genetic counselling into healthcare. This has garnered more attention over the years as genetic testing becomes more commonplace but, regardless of the setting in which it is offered, it will be important for psychiatric genetic counselling to be delivered in an evidence-based manner [11] and will require integration into their professional training.

## Conclusion

Psychiatric genetic counselling holds great potential for benefiting children and adolescents experiencing mental health disorders, as well as their parents/caregivers. While children and adolescents want to better understand the cause of their disorder/s, little empirical research has been conducted on the communication of genetic information to this distinct patient group. Therefore, we propose that investigating how to best address psychiatric disorders and deliver genetic information to children and adolescents must become a research priority in order to support and inform evidence-based practice and guidelines.
